# Supplementary material for: Wetlands set the pace of annual runoff in the northern Great Plains
Source: Commun Earth Environ. 2026 Mar 11;7(1):368. doi: 10.1038/s43247-026-03318-0 (PMC13111149; doi:10.1038/s43247-026-03318-0)
Supplement: Supplementary file 2 — Supplementary Information of “Wetlands Set the Pace of Annual Runoff in the Northern Great Plains” [file 43247_2026_3318_MOESM2_ESM.pdf]

Supplementary Information of "Wetlands Set the Pace of Annual Runoff in the Northern Great Plains"

Javad Rahmani[1], Irena F. Creed[2] , Pascal Badiou[3] and Ali A Ameli[1]*

[1]Department of Earth, Ocean & Atmospheric Sciences, University of British Columbia, Vancouver, Canada
[2]Department of Physical & Environmental Sciences, University of Toronto, Toronto, Canada
[3]Institute for Wetland and Waterfowl Research, Ducks Unlimited Canada, Manitoba, Canada

*Corresponding Author: Ali A. Ameli

Supplementary Note 1:
Do Common Climatic Drivers Control Both Annual ROR and Annual MIWA?

The stronger partial correlations observed between MIWA and runoff ratio (ROR), relative to those between climate drivers and ROR, suggest that the climate-centric framework—while valuable—cannot fully account for interannual runoff variability in PPR catchments. While MIWA strongly controls changes in ROR in most study catchments, it is plausible that shared climatic factors modulate both. We explored this possibility through five hypotheses:

*Hypothesis 1: Extreme Rainfall Controls Both MIWA and ROR***.** Across all catchments, the correlation between the 95th percentile of annual rainfall and ROR is weak and non-significant (median Spearman $\rho = 0.25$; mean = 0.24; Figure 2a). Similarly, its correlation with MIWA is also negligible (median = 0.17; mean = 0.16; Supplementary Figure 3g). Correlations involving annual maximum daily rainfall yielded similarly low values for both ROR (median = 0.16; mean = 0.18) and MIWA (median = 0.08; mean = 0.10; Supplementary Figures 3h). These findings indicate that extreme rainfall, as captured by standard intensity metrics, does not significantly influence the interannual variability of either MIWA or ROR, and thus is unlikely to be a confounding common driver.

Hypotheses 2 & 3: Snow Fraction and/or Snow Persistence Control Both MIWA and ROR. Snow fraction (SF) showed weak correlations with ROR (median $\rho = 0.23$; mean $\rho = 0.23$; Figure 2a). In contrast, snow persistence (SP)—a metric reflecting the duration of snowpack presence—demonstrated a substantially stronger relationship with ROR (median = 0.52; mean = 0.48; Figure 2a). This was particularly evident in catchments across North Dakota, South Dakota, Iowa, and Minnesota. However, even in catchments where the SP–ROR correlation was high (e.g., 05053000, 05059700, 05051300), MIWA–ROR correlations were much higher (Supplementary Figure 4d-f). Moreover, positive SP–MIWA correlations in these same catchments (Supplementary Figure 4d-f), and across several other catchments (Supplementary Figure 3a) suggest a mediating role of inundation. Partial correlation analysis across all catchments supports this mediation hypothesis. When controlling for SP, the partial correlation between MIWA and ROR remains high (median = 0.63; mean = 0.60; Figure 2b). Conversely, controlling for MIWA reduces the SP–ROR correlation to a median of 0.28 (mean = 0.25). This indicates that while SP influences annual ROR, its effect operates largely through its impact on MIWA—by modulating the extent and duration of wetland inundation, which enhances catchment connectivity and runoff efficiency.

Hypothesis 4: Current-year aridity influences both MIWA and ROR. Current-year aridity showed moderately large correlations with ROR (median = –0.52; mean = –0.44; Figure 2a), particularly across catchments in Iowa, South Dakota, North Dakota, and Minnesota. However, in these same catchments, MIWA–ROR correlations were generally stronger (Figure 2a),

indicating that aridity alone does not fully explain interannual runoff variability. Partial correlation analysis reinforces this interpretation. When controlling for MIWA, the aridity–ROR correlation decreases markedly (median = –0.32; mean = –0.28; Figure 2b), whereas conditioning on current-year aridity leaves the MIWA–ROR partial correlation essentially unchanged (median = 0.67; mean = 0.62; Figure 2b). This asymmetry indicates that current-year aridity primarily influences ROR indirectly, through its effect on annual wetland inundation extent, rather than exerting a strong direct control on runoff generation.

Hypothesis 5: Lagged Aridity Influences Both MIWA and ROR. Of three lag structures tested (previous-year aridity, average of current and previous year aridity, and average of two previous years aridity), previous-year aridity (PY-aridity) yielded the strongest association with ROR, showing a median (mean) Spearman correlation of –0.40 (–0.40) across catchments (Figure 2a). In contrary to concurrent-year aridity that shows strong correlations with ROR primarily in catchments across the U.S. portion of the PPR, the PY-aridity effect surpasses that of concurrent-year aridity in fill–spill dominated regions of Manitoba, and eastern Saskatchewan (Compare Figures 2a & 3). Example catchments (05JE006, 05NG001, 05MH005) further illustrate that the magnitude of MIWA–ROR correlations exceeds that of PY-aridity–ROR (Supplementary Figure 4a-c), and moderately large negative correlations between PY-aridity and MIWA in 05JE006 (Supplementary Figure 4a) suggest a causal chain: wetter antecedent conditions (lower PY-aridity) lead to greater current-year inundation, enhancing connectivity and elevating ROR. Partial correlations reinforce this mediation. When controlling for PY-aridity, the MIWA–ROR link remains strong (median = 0.66; mean = 0.60), but when controlling for MIWA, the PY-aridity–ROR correlation falls sharply (median = –0.21; mean = –0.22) (Figure 2b). This pattern confirms that antecedent aridity primarily influences ROR by shaping seasonal inundation dynamics, not through a direct hydrologic mechanism.

Hypothesis 6: Intra-annual (seasonal) water availability is the common driver of MIWA and ROR. April NWI (NWI_{April}) exhibited only weak correlations with ROR (median $\rho = 0.28$; mean $\rho = 0.27$; Figure 2a). By contrast, maximum monthly NWI (NWI_{MAX}) showed stronger correlations (median $\rho = 0.54$; mean $\rho = 0.49$; Figure 2a), particularly in some catchments across Iowa, South Dakota, Minnesota, North Dakota, and Manitoba. However, once controlling for MIWA, the NWI_{MAX}–ROR correlation drops substantially (median $\rho = 0.38$; mean $\rho = 0.35$; Figure 2b), whereas adjusting for NWI_{MAX}, the MIWA–ROR association remained strong (median $\rho = 0.64$; mean $\rho = 0.60$; Figure 2b).

Overall, among the 75 fill-spill dominated catchments, SP emerged as the primary determinant of MIWA in 37 basins (median $\rho = 0.63$), while PY-aridity in 2, current-year aridity in 3 and NWI_{MAX} in 3 basins were the determinant (Supplementary Figure 5). In the remainder, other currently unquantified factors likely govern annual inundation variability. These results highlight the importance of snowmelt-related controls on wetland hydrology.

Supplementary Table

Supplementary Table 1| Spatial (catchment-to-catchment) correlation between static physio-climatic attributes and parameter *b*, which reflects the degree of nonlinearity in the functional form of the MIWA–ROR relationship.

Each attribute was evaluated as a potential control on the nonlinearity parameter (*b*) in the power-law relationship between MIWA and ROR. Long-term average climatic metrics were calculated as the average values over the 38-year study period. Data sources and calculation methods are detailed in the Data Section of the main text.

Potential Driver	Unit	Spearman Correlation	p-value
GIWs Maximum Inundated Extent	%	0.65	2.60E-10
Total Wetlands Maximum Inundated Extent	%	0.62	2.39E-09
Riparian Wetlands Maximum Inundated Extent	%	0.50	5.05E-06
Aridity	-	0.42	0.00016
Elevation	m	0.41	0.00025
Snow fraction	-	0.41	0.00027
Soil Moisture	-	-0.35	0.00189
Snow Presence	-	0.33	0.00379
Seasonality index	-	0.32	0.00493
LULC Grassland Fraction	%	0.31	0.00638
River density	m/m^2	0.27	0.01983
Water Table Depth	m	0.26	0.02593
LULC Cropland Fraction	%	-0.24	0.04092
HAND	m	0.21	0.07725
Area	m^2	0.19	0.10318
LULC Urban Fraction	%	-0.15	0.0144
Slope	%	0.13	0.6114
Soil Clay Content	-	0.10	0.5416
Soil Sand Content	-	-0.07	0.7277
LULC Forest Fraction	-	0.06	0.7788
Soil Silt Content	-	-0.01	0.8577

Supplementary Figures

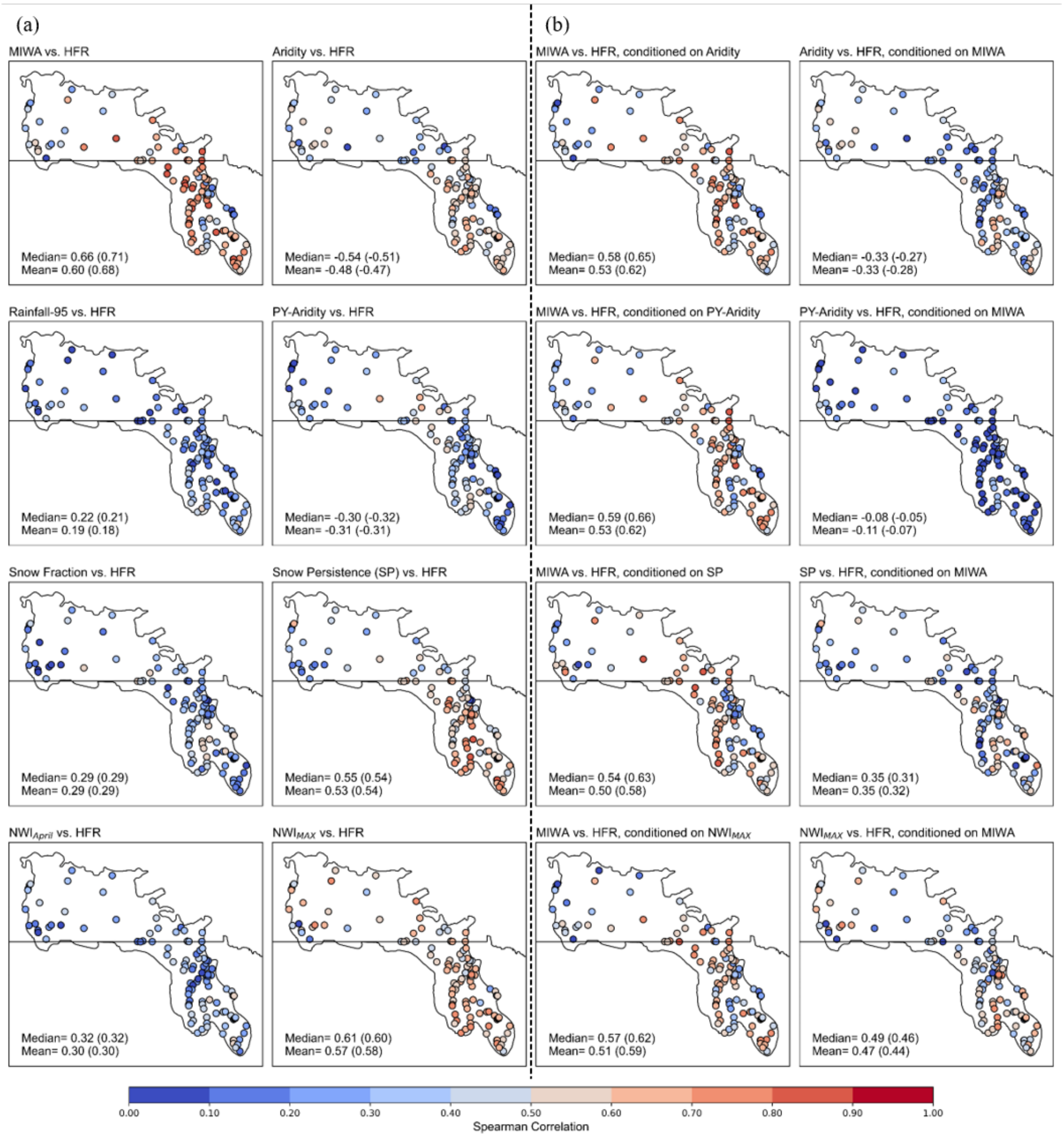

Supplementary Figure 1 | Climatic and landscape controls on interannual variability in the high-flow ratio (HFR). Full and partial correlations between annual HFR and climatic (and landscape) drivers across all 109 study catchments. To enable consistent comparison, color maps display the absolute values (magnitudes) of correlation coefficients. Median and mean correlation values are reported across all catchments, with values in parentheses denoting statistics for the 75 fill–spill dominated catchments. (a) Full Spearman correlations between HFR and each driver: maximum inundated wetland area (MIWA), current-year aridity index, 95th percentile of rainfall ($Rainfall_{95}$), previous-year aridity (PY-aridity), snow fraction (SF), snow persistence (SP), April NWI (NWI_{April}), and maximum monthly NWI in each year (NWI_{MAX}). (b) Partial correlations between HFR and MIWA, controlling for each of the four main confounders—aridity index, PY-aridity, SP, and NWI_{MAX}—individually. Partial correlation analysis was not conducted for SF, $Rainfall_{95}$, and April NWI due to their weak associations with HFR in the full correlation results. All figure elements are original; the figure was generated in Python.

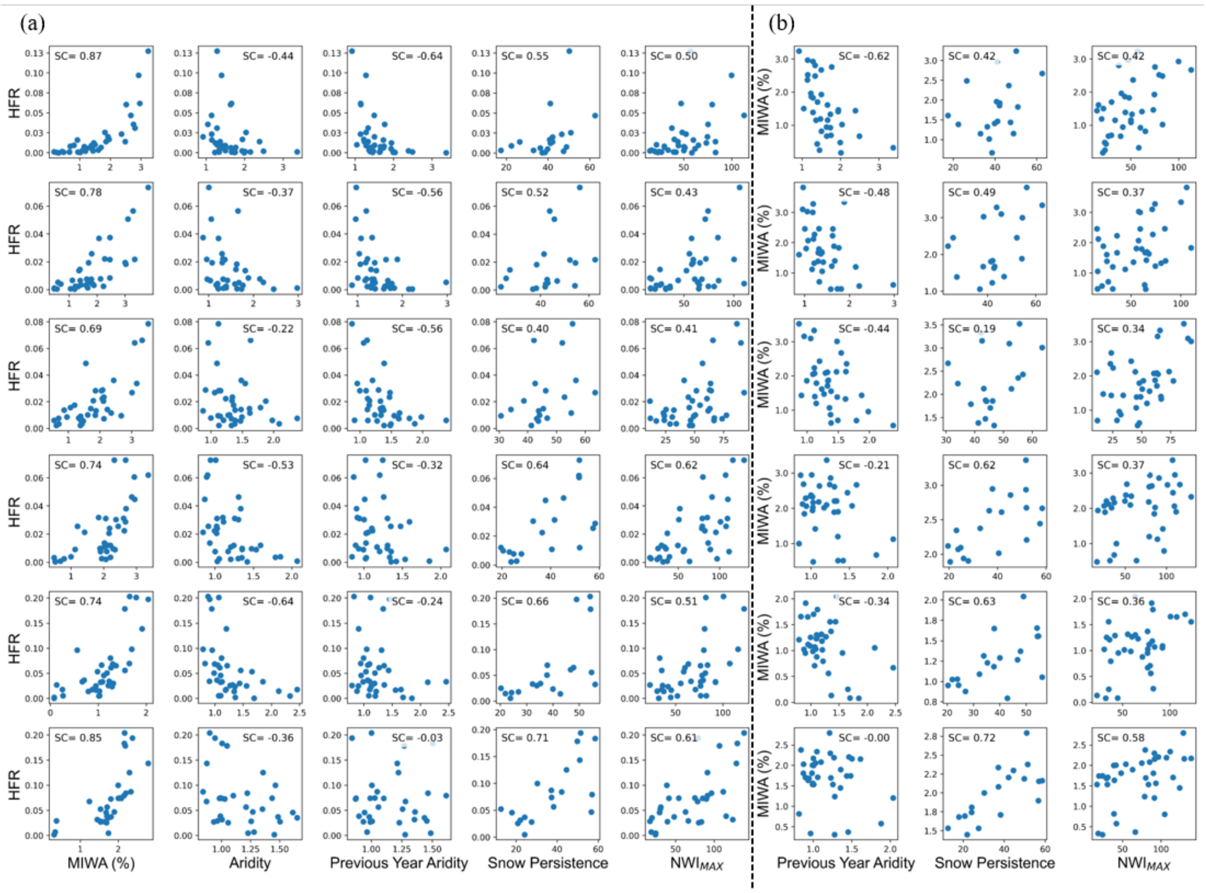

Supplementary Figure 2 | Scatterplot analysis of interannual variability in high-flow ratio (HFR) and its potential climatic and landscape drivers for six example fill-spill dominated catchments.

Each row corresponds to one catchment, ordered top to bottom by station code: 05JE006 (Saskatchewan), Manitoba (05NG001), 05MH005 (Manitoba), 05053000 (North Dakota), 05059700 (North Dakota), and 05051300 (Minnesota). (a) Relationships between annual HFR and each of five drivers: maximum inundated wetland area (MIWA), aridity index, previous-year aridity index (PY-aridity), snow persistence (SP), and maximum monthly NWI in each year (NWI_{MAX}). (b) Moderately strong association between MIWA and PY-aridity (uppermost catchment) and between MIWA and SP (bottom three catchments)—highlighting potential mediating effects. Spearman correlation (SC) coefficients are reported for each panel. These six fill-spill catchments correspond to those illustrated in Figure 4 of the main text and Supplementary Figure 4. All figure elements are original; the figure was generated in Python.

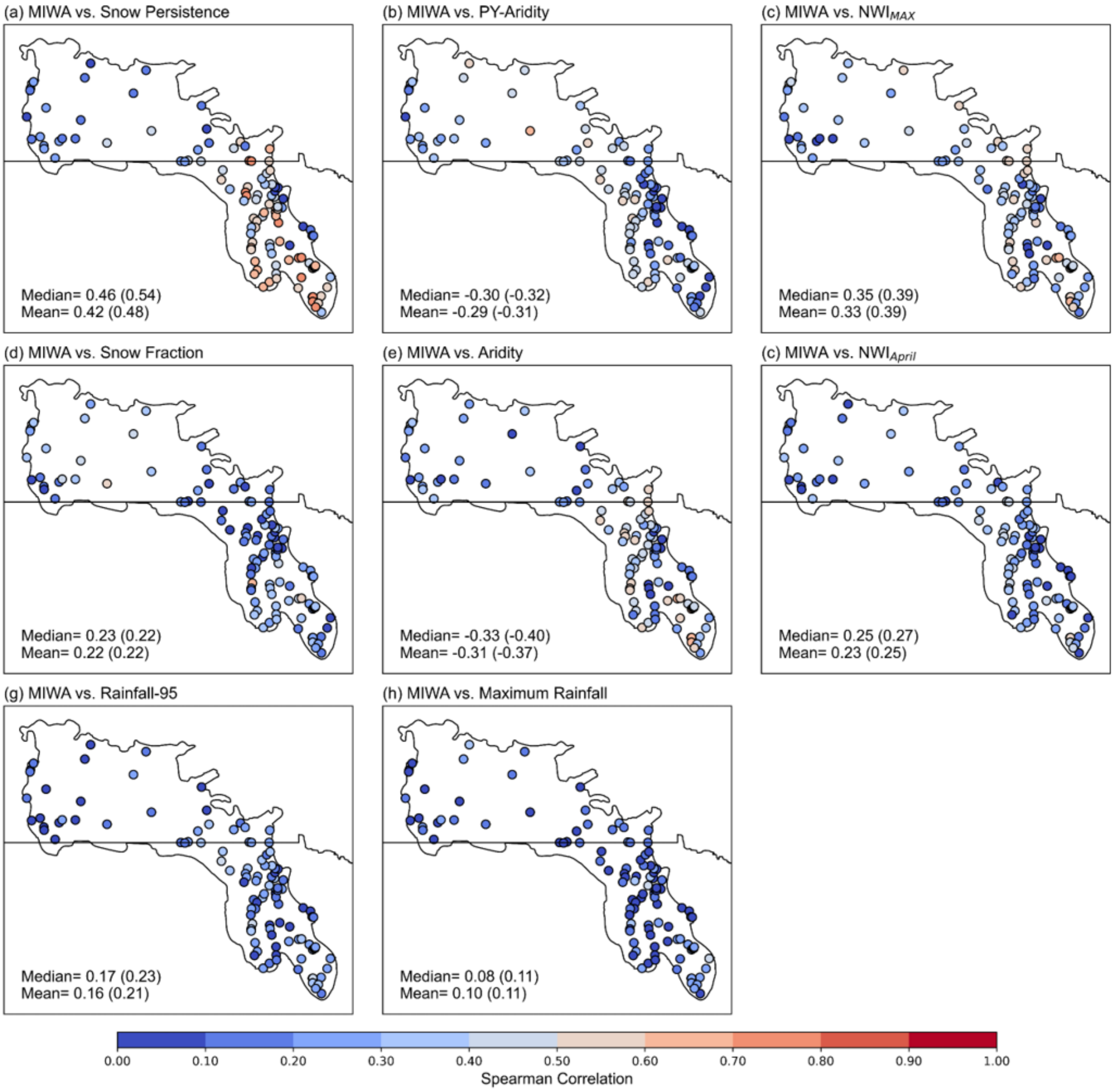

Supplementary Figure 3 | Climatic drivers of annual maximum inundated wetland area (MIWA). Spearman correlations between MIWA and potential climatic drivers are shown across 109 study catchments. Climate drivers include snow persistence (SP), previous-year aridity (PY-aridity), maximum monthly net water input (NWI_{MAX}), snow fraction (SF), aridity index, April NWI (NWI_{April}), 95th percentile of rainfall ($Rainfall_{95}$), and maximum rainfall at the single wettest day each year. To facilitate comparison, the color map displays the absolute values of correlation coefficients. Median and mean correlation values are reported for all catchments, with values in parentheses denoting statistics for the 75 fill–spill dominated catchments. Seasonality index showed negligible correlation with MIWA in all study catchments and hence was not shown here. All figure elements are original; the figure was generated in Python.

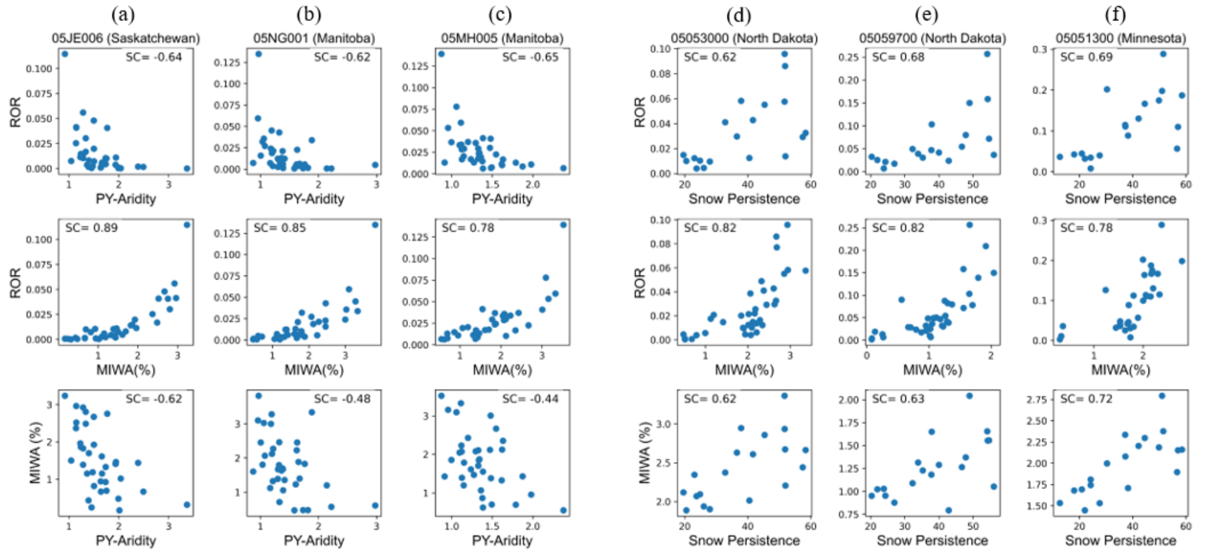

Supplementary Figure 4 | Scatterplot analysis of interannual variabilities in runoff ratio (ROR) and MIWA and their potential climatic drivers for six example fill-spill dominated catchments.

Panels (a–c) illustrate catchments where previous-year aridity (PY-aridity) appears to influence the runoff ratio (ROR) indirectly through its effect on the maximum inundated wetland area (MIWA). In each catchment, although PY-aridity is correlated with ROR, the correlation between MIWA and ROR is even stronger. Moreover, PY-aridity shows a moderate to large correlation with MIWA, reinforcing the hypothesis that MIWA mediates the relationship between PY-aridity and ROR. Similarly, panels (d–f) highlight catchments where snow persistence (SP) influences the runoff ratio (ROR), potentially through its control on the MIWA. In all three cases, while SP shows a strong correlation with ROR, the correlation between MIWA and ROR is notably stronger. Furthermore, SP is positively correlated with MIWA, suggesting that increased snow persistence enhances wetland inundation. These patterns support the role of MIWA as a mediator in the relationship between SP and ROR. These six selected catchments correspond to those illustrated in Figure 4 of the main text and Supplementary Figure 2. All figure elements are original; the figure was generated in Python.

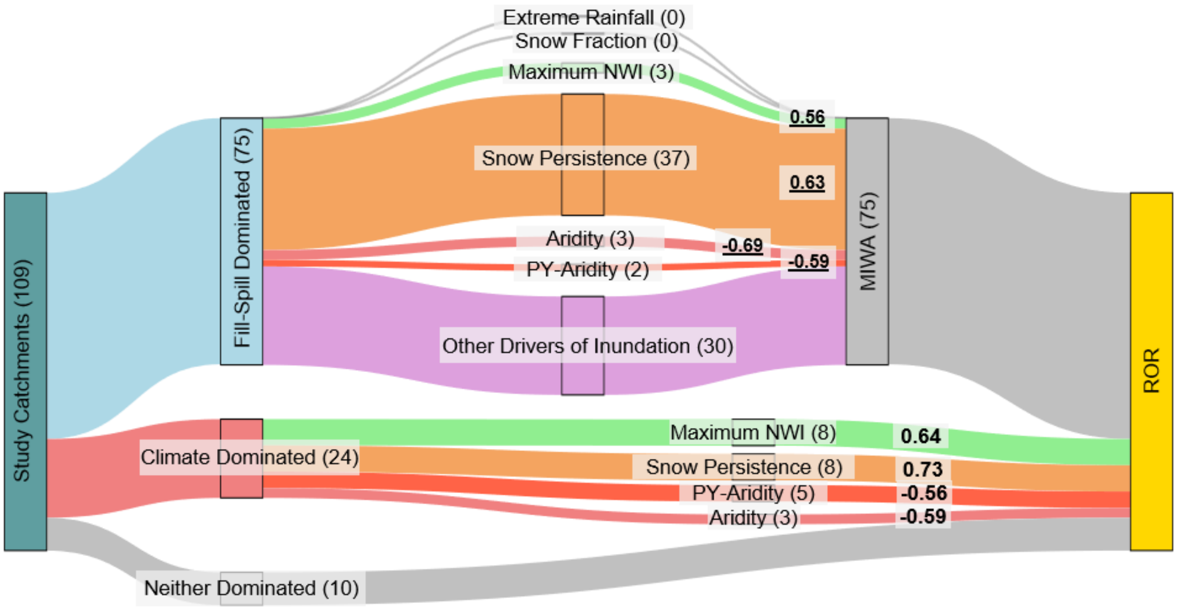

Supplementary Figure 5 | Conceptual synthesis of the mediating role of wetland inundation extent in transmitting climatic signals.

Schematic summarizing the strength and direction of interannual relationships between climatic drivers, annual maximum inundated wetland area (annual MIWA), and runoff ratio (ROR) across fill–spill dominated catchments, and between climatic drivers and runoff ratio (ROR) across climate dominated catchments. The number in parentheses and the thickness of each connecting segment indicate the number of catchments represented in each path. A total of 75 catchments were classified as fill–spill dominated and 24 as climate-dominated (see Method Section for classification details). Within the fill–spill dominated group, the number in parentheses beside each climate driver—and the corresponding segment thickness—reflects the number of catchments where that climate variable has a Spearman correlation with MIWA exceeding 0.5 and is stronger than the correlation of the alternative climate driver with MIWA. Catchments not meeting either condition are grouped under "Other Drivers of Inundation". In 60% (45) of fill-spill dominated catchments, one climate driver controls MIWA, and in the remaining 40% (30) of fill-spill dominated catchments, none of the climatic drivers used in our study strongly associate with MIWA. Bold and underlined numbers represent the median Spearman correlation between each climatic variable within each path and MIWA. In 49% of fill–spill dominated catchments, snow persistence emerges as the dominant driver of MIWA—exhibiting the highest correlation with MIWA among all climatic drivers and exceeding $\rho = 0.5$ (median correlation = 0.63). In the Climate-dominated group, the number in parentheses beside each climate driver—and the corresponding segment thickness—reflects the number of catchments where that climate variable has a Spearman correlation with ROR exceeding 0.5 and is stronger than the correlation of the alternative climate driver with ROR. Bold numbers represent the median Spearman correlation between each climatic variable within each path and ROR. All figure elements are original.

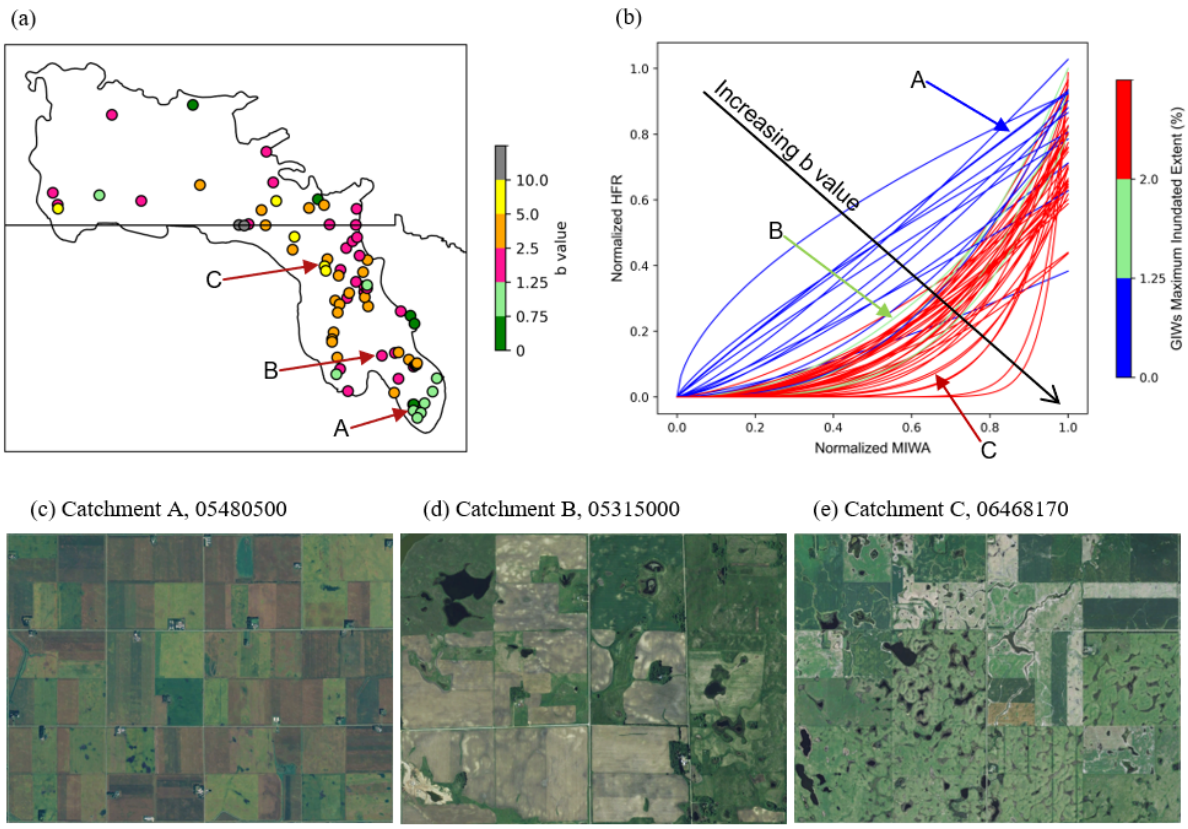

Supplementary Figure 6 | Functional analysis of the relationship between maximum inundated wetland area (MIWA) and high-flow ratio (HFR) in 75 fill–spill dominated catchments.
(a) Spatial distribution of the power-law nonlinearity parameter (*b*) across catchments. Values of $b > 1$ indicate threshold-like “buffer” behavior, where HFR remains low until a critical inundation threshold is surpassed. Values near 1 reflect a linear response, and $b < 1$ corresponds to plateau behavior, where HFR increases rapidly at low MIWA but saturates thereafter. (b) Fitted power-law curves for catchments with $R^2 > 0.5$. As *b* increases, the curve shapes shift from concave-down to concave-up, signaling a transition from plateau to buffered runoff regimes. The color scale represents the long-term maximum inundation extent of geographically isolated wetlands (GIWs), a key determinant of the b-value. (c–e) Aerial imagery from three representative catchments with contrasting *b* values and GIW extents. (c) Catchment A (Iowa): $b = 0.96$, GIWs extent = 0.73%. (d) Catchment B (Minnesota): $b = 2.09$, GIWs extent = 1.38%. (e) Catchment C (North Dakota): $b = 5.45$, GIWs extent = 3.21%. These three catchments correspond to those illustrated in Figure 5 of the main text. Panels a–b were generated in Python. Panels c–e: Map data ©2024–2025 Google. Imagery ©2024–2025 Airbus.
