# Supplementary material for: Wetlands set the pace of annual runoff in the northern Great Plains
Source: Commun Earth Environ. 2026 Mar 11;7(1):368. doi: 10.1038/s43247-026-03318-0 (PMC13111149; doi:10.1038/s43247-026-03318-0)
Supplement: Supplementary file 3 — Reporting summary [file 43247_2026_3318_MOESM3_ESM.pdf]

nature portfolio

Corresponding author(s): Ali Ameli

Last updated by author(s): Oct 10, 2025

Reporting Summary

Nature Portfolio wishes to improve the reproducibility of the work that we publish. This form provides structure for consistency and transparency in reporting. For further information on Nature Portfolio policies, see our Editorial Policies and the Editorial Policy Checklist.

Statistics

For all statistical analyses, confirm that the following items are present in the figure legend, table legend, main text, or Methods section.

n/a	Confirmed	
☐	☒	The exact sample size (*n*) for each experimental group/condition, given as a discrete number and unit of measurement
☐	☒	A statement on whether measurements were taken from distinct samples or whether the same sample was measured repeatedly
☐	☒	The statistical test(s) used AND whether they are one- or two-sided<br>*Only common tests should be described solely by name; describe more complex techniques in the Methods section.*
☐	☒	A description of all covariates tested
☐	☒	A description of any assumptions or corrections, such as tests of normality and adjustment for multiple comparisons
☐	☒	A full description of the statistical parameters including central tendency (e.g. means) or other basic estimates (e.g. regression coefficient) AND variation (e.g. standard deviation) or associated estimates of uncertainty (e.g. confidence intervals)
☐	☒	For null hypothesis testing, the test statistic (e.g. *F*, *t*, *r*) with confidence intervals, effect sizes, degrees of freedom and *P* value noted<br>*Give P values as exact values whenever suitable.*
☒	☐	For Bayesian analysis, information on the choice of priors and Markov chain Monte Carlo settings
☒	☐	For hierarchical and complex designs, identification of the appropriate level for tests and full reporting of outcomes
☐	☒	Estimates of effect sizes (e.g. Cohen's *d*, Pearson's *r*), indicating how they were calculated

Our web collection on statistics for biologists contains articles on many of the points above.

Software and code

Policy information about availability of computer code

Data collection	Datasets were obtained from open repositories — ERA5-Land (ECMWF CDS), MODIS Snow Cover, Global Surface Water (Pekel et al., 2016), HYDAT and USGS NWIS.
Data analysis	The executable code used to generate results is available at: https://github.com/j-rahmani/Pothole-Inundation. The repository includes a README file with detailed instructions for installing the required libraries and running the code in Python. In addition, the expected outputs of the code—covering most of the figures used in the main manuscript and supplementary document—are provided for comparison.

For manuscripts utilizing custom algorithms or software that are central to the research but not yet described in published literature, software must be made available to editors and reviewers. We strongly encourage code deposition in a community repository (e.g. GitHub). See the Nature Portfolio guidelines for submitting code & software for further information.

Data

Policy information about availability of data

All manuscripts must include a data availability statement. This statement should provide the following information, where applicable:

- Accession codes, unique identifiers, or web links for publicly available datasets
- A description of any restrictions on data availability
- For clinical datasets or third party data, please ensure that the statement adheres to our policy

All data sources used in this study are cited in the main text. Processed inter-annual and intra-annual datasets—including MIWA, aridity, previous year aridity, snow

persistence, snow fraction, seasonality, April NWI, maximum monthly NWI, maximum Rainfall, 95th percentile of Rainfall, 95th percentile of precipitation, 95th percentile of Streamflow, ROR, and HFR—are available at: https://zenodo.org/records/18153132. The package includes a README that documents each dataset.

Research involving human participants, their data, or biological material

Policy information about studies with human participants or human data. See also policy information about sex, gender (identity/presentation), and sexual orientation and race, ethnicity and racism.

Reporting on sex and gender	*Use the terms sex (biological attribute) and gender (shaped by social and cultural circumstances) carefully in order to avoid confusing both terms. Indicate if findings apply to only one sex or gender; describe whether sex and gender were considered in study design; whether sex and/or gender was determined based on self-reporting or assigned and methods used. Provide in the source data disaggregated sex and gender data, where this information has been collected, and if consent has been obtained for sharing of individual-level data; provide overall numbers in this Reporting Summary. Please state if this information has not been collected. Report sex- and gender-based analyses where performed, justify reasons for lack of sex- and gender-based analysis.*
Reporting on race, ethnicity, or other socially relevant groupings	*Please specify the socially constructed or socially relevant categorization variable(s) used in your manuscript and explain why they were used. Please note that such variables should not be used as proxies for other socially constructed/relevant variables (for example, race or ethnicity should not be used as a proxy for socioeconomic status). Provide clear definitions of the relevant terms used, how they were provided (by the participants/respondents, the researchers, or third parties), and the method(s) used to classify people into the different categories (e.g. self-report, census or administrative data, social media data, etc.) Please provide details about how you controlled for confounding variables in your analyses.*
Population characteristics	*Describe the covariate-relevant population characteristics of the human research participants (e.g. age, genotypic information, past and current diagnosis and treatment categories). If you filled out the behavioural & social sciences study design questions and have nothing to add here, write "See above."*
Recruitment	*Describe how participants were recruited. Outline any potential self-selection bias or other biases that may be present and how these are likely to impact results.*
Ethics oversight	*Identify the organization(s) that approved the study protocol.*

Note that full information on the approval of the study protocol must also be provided in the manuscript.

Field-specific reporting

Please select the one below that is the best fit for your research. If you are not sure, read the appropriate sections before making your selection.

☐ Life sciences ☐ Behavioural & social sciences ☒ Ecological, evolutionary & environmental sciences

For a reference copy of the document with all sections, see nature.com/documents/nr-reporting-summary-flat.pdf

Ecological, evolutionary & environmental sciences study design

All studies must disclose on these points even when the disclosure is negative.

Study description	This quantitative large-sample analysis examines how annual wetland inundation extent controls interannual variability in runoff across the Prairie Pothole Region (PPR) of North America. The study integrates 38 years (1984–2021) of satellite-derived wetland inundation maps, hydroclimatic data, and streamflow from 109 unregulated catchments to disentangle the roles of climate and wetland connectivity in regulating annual runoff ratio and high-flow response.
Research sample	The research sample consists of 109 gauged catchments distributed across the Canadian and U.S. portions of the PPR, ranging in size from hundreds to tens of thousands of square kilometers. Each catchment represents a natural hydrologic unit characterized by variable wetland density and connectivity. Data sources include publicly available streamflow archives (HYDAT, USGS NWIS), ERA5-Land reanalysis, MODIS snow persistence, and Landsat-based Global Surface Water datasets.
Sampling strategy	Sample size was determined by the availability of unregulated catchments with ≥20 years of complete hydrometeorological data (1984–2021). No predetermined sample-size calculation was required. The number of catchments ensures broad climatic and physiographic representation across the PPR and provides sufficient statistical power to detect dominant climatic versus wetland controls.
Data collection	All data were obtained from open-access repositories. Streamflow data were downloaded from USGS and Environment and Climate Change Canada. Climate data (precipitation, temperature, potential evapotranspiration) were retrieved from ERA5-Land and the Global Land Surface PET dataset. Snow persistence was derived from MODIS/Terra 8-Day L3 products, and wetland inundation from the Landsat-based Global Surface Water dataset via Google Earth Engine. No new measurements were collected.
Timing and spatial scale	The analysis spans 1984–2021 at annual resolution, with some subannual (monthly) metrics derived for snowmelt and net water input. Data were aggregated by calendar year. The spatial scale is catchment-based, covering 109 drainage basins distributed across ~780,000 km² of the Prairie Pothole Region. Temporal alignment analyses also used monthly data to evaluate seasonality.

Data exclusions	Catchments were excluded if they were located immediately downstream of dams or had <20 years of data with ≥95% annual completeness. No other data were excluded. Exclusion criteria were pre-established to ensure unregulated hydrologic conditions and data consistency across all analyses.
Reproducibility	All analyses were reproduced using the publicly available Python scripts and datasets in the GitHub repository https://github.com/j-rahmani/Pothole-Inundation. Replication produced identical results and figures to those in the manuscript and Supplementary Information, confirming full reproducibility.
Randomization	Not applicable. No experimental treatments or group allocations were conducted. All analyses were observational, based on fixed catchments and environmental datasets.
Blinding	Not applicable. Data processing and analysis involved automated computational workflows applied uniformly to all catchments. No subjective classification or human scoring requiring blinding was performed.

Did the study involve field work? ☐ Yes ☒ No

Reporting for specific materials, systems and methods

We require information from authors about some types of materials, experimental systems and methods used in many studies. Here, indicate whether each material, system or method listed is relevant to your study. If you are not sure if a list item applies to your research, read the appropriate section before selecting a response.

Materials & experimental systems

n/a	Involved in the study
☒	☐ Antibodies
☒	☐ Eukaryotic cell lines
☒	☐ Palaeontology and archaeology
☒	☐ Animals and other organisms
☒	☐ Clinical data
☒	☐ Dual use research of concern
☒	☐ Plants

Methods

n/a	Involved in the study
☒	☐ ChIP-seq
☒	☐ Flow cytometry
☒	☐ MRI-based neuroimaging

Plants

Seed stocks	Not applicable. No plant specimens or seed stocks were collected, cultivated, or analyzed in this study. The research relies entirely on remotely sensed and modeled hydrological data.
Novel plant genotypes	Not applicable. The study did not involve any plant materials, transgenic or gene-edited lines, mutagenesis, or hybridization experiments.
Authentication	Not applicable. No plant materials or genotypes were used; therefore, authentication or validation procedures were not required.
